# A biopsy-validated study of combined DL-upscaled MRSI and (18F)-FACBC PET for presurgical glioma diagnosis

**DOI:** 10.3389/fonc.2026.1968798

**Published:** 2026-09-30

**Authors:** Erin Beate Bjørkeli, Anna Maria Karlberg, Benedikte Emilie Vindstad, Lars Kjelsberg Pedersen, Ole Skeidsvoll Solheim, Jonn Terje Geitung, Morteza Esmaeili, Live Eikenes

**Affiliations:** 1 Department of Diagnostic Imaging, Akershus University Hospital, Lørenskog, Norway; 2 Institute of Clinical Medicine, University of Oslo, Oslo, Norway; 3 Department of Circulation and Medical Imaging, Norwegian University of Science and Technology (NTNU), Trondheim, Norway; 4 Department of Radiology and Nuclear Medicine, St. Olavs Hospital, Trondheim University Hospital, Trondheim, Norway; 5 Department of Neurosurgery, Ophthalmology and Otorhinolaryngology, University Hospital of North Norway, Tromsø, Norway; 6 Department of Neurosurgery, St. Olavs Hospital, Trondheim University Hospital, Trondheim, Norway; 7 Department of Neuroscience, Norwegian University of Science and Technology, Trondheim, Norway; 8 Department of Electrical Engineering and Computer Science, University of Stavanger, Stavanger, Norway

**Keywords:** deep learning, fluciclovine PET molecular imaging, glioblastoma, metabolite MRI, multimodal imaging

## Abstract

Medical imaging is crucial for glioma management. Combined with MRI, amino acid PET may improve glioma diagnosis, biopsy targeting, and tumor delineation compared to structural MRI alone. Magnetic resonance spectroscopic imaging (MRSI) complements both structural MRI and PET by detecting metabolites such as N-acetylaspartate (NAA), creatine (Cr), and choline (Cho), which are markers for brain health and tumor malignancy, but is challenged by low spatial resolution. This study evaluates whether upscaled MRSI enhanced by deep learning can improve diagnostic accuracy and serve as a complement or alternative to PET for glioma classification. Ten glioma patients (ages 24-80) were included. Presurgical [18F]-FACBC PET/MRI, including proton 2D MRSI, was acquired for all patients. Thirty-two image-guided biopsies were sampled during surgery from the patients and classified as glioma tissue or non-tumor tissue, and according to *IDH1* status. For each biopsy location, tumor-to-background ratio (TBR) and standardized uptake value (SUV) from PET, and tCho/NAA and tCho/tCr ratios from MRSI were calculated. ROC analysis was used to assess the accuracy of [18F]-FACBC PET and upscaled MRSI, and the combinations of these in classifying glioma vs non-tumor tissue and *IDH1* status. Among the individual biomarkers evaluated, the tCho/NAA ratio from the deep learning-based model demonstrated the highest apparent AUC for classifying glioma vs. non-tumor tissue (AUC = 0.87, 95% CI: 0.66-1.0), compared with tCho/tCr (AUC = 0.81, 95% CI: 0.54-1:00), SUV (AUC = 0.71, 95% CI: 0.49-0.90), and TBR (AUC = 0.68, 95% CI: 0.48-0.86). Combining TBR with tCho/NAA and/or tCho/tCr improved tissue classification compared to either modality alone, where TBR+tCho/NAA+tCho/tCr showed the best results (AUC = 0.91, 95% CI: 0.71-1.0). MRSI was a poor predictor for *IDH1*-status (tCho/NAA: AUC = 0.67, 95% CI: 0.44-0.88 and tCho/tCr: AUC = 0.38, 95% CI: 0.17-0.60), while PET was an excellent predictor (SUV: AUC = 0.83, 95% CI: 0.66-0.95 and TBR: AUC = 0.82, 95% CI: 0.65-0.94) and the combination of SUV, tCho/NAA, and tCho/tCr was a best predictor (AUC = 0.96, 95% CI: 0.88-1.0). Combining DL-upscaled MRSI with [18F]-FACBC PET appeared to enhance discrimination between glioma and non-tumor tissue, a finding that warrants validation in larger, prospective studies.

## Introduction

Adult diffuse gliomas are classified according to the 2021 World Health Organization (WHO) classification of tumors of the central nervous system (CNS) into glioblastoma *IDH*-wild-type (grade 4), astrocytoma *IDH*-mutant (grades 2–4), and oligodendroglioma *IDH*-mutant with 1p/19q co-deletion (grades 2–3) (1). Gliomas are highly heterogeneous, with regions displaying different properties and degrees of malignancy (2, 3). Presurgical imaging is important for tumor delineation, predicting diagnoses, and surgical planning.

Structural magnetic resonance imaging (MRI) is the imaging method of choice for brain tumor examinations due to its high-resolution soft tissue contrast, but may have limitations when it comes to identifying the most malignant regions. Metabolite imaging is a group of diagnostic techniques that provide non-invasive visualization of metabolic activity in living tissues and can be visualized by either positron emission tomography (PET) or magnetic resonance spectroscopy imaging (MRSI). Alterations in metabolic activity can be indicative of underlying pathologies and may exhibit both the presence and aggressiveness of tumors (4). Metabolic imaging can therefore aid in identifying the most aggressive regions of the tumor. MRS and PET are often used in combination with MRI techniques to complement anatomical imaging.

Glioma cells exhibit increased transmembrane amino acid transport to facilitate enhanced protein synthesis. The amino acid PET tracer 18F-fluorocyclobutane-1-carboxylic acid ([18F]-FACBC) takes advantage of this mechanism, utilizing amino acid transporters for cellular uptake. Previous studies using [18F]-FACBC PET in gliomas demonstrated its utility in distinguishing tumors from normal brain tissue and in correlating uptake patterns with histopathological findings. In comparison with MRI performed according to acknowledged standards for examining gliomas, [18F]-FACBC PET may improve diagnostic accuracy in identifying tumor infiltration beyond visible abnormalities on structural MRI (5–8). This highlights its potential role in guiding neurosurgery and therapeutic planning by delineating metabolically active tumor regions more effectively than structural MRI.

Complementary to this, MRSI provides information on the tissue content of specific metabolites, such as N-acetyl aspartate (NAA), creatine+phosphocreatine (Cr+PCr refered to as tCr), and choline-containing metabolites (tCho) (9, 10). These metabolites are present in lower concentrations compared to the dominant water and lipid signals, typically in the range of a few millimoles per liter (mM) within the brain tissue (11). The concentrations may vary in different brain regions and can also be influenced by factors such as age, health status, and metabolic activity (12–14). In gliomas, decreased NAA indicates neuronal loss, whereas elevated tCho reflects increased membrane turnover; thus, elevated tCho/tCr and tCho/NAA ratios serve as key biomarkers for tumor detection (15) and treatment evaluations (16). These variations make the metabolites and their ratios valuable markers for brain examinations. However, MRSI is limited by its lower spatial resolution compared to structural MRI (normally 1 mm isotropic resolution). Signal averaging or the use of larger voxel volumes is essential to enhance the signal-to-noise ratio (SNR) in MRSI (17). However, increasing voxel size reduces spatial resolution in MRSI sequences, and typically the spatial resolution is limited from 16×16 to 64×64 voxels. For instance, a standard 32×32 matrix covering the adult brain with a field-of-view of 220 mm (2) yields a nominal voxel size of approximately 6.875×6.875 mm (2). Even with advanced research protocols, MRSI spatial resolution remains coarser than other MR modalities. Post-processing techniques have been developed for higher resolution MRSI, but many of these methods encounter challenges such as high complexity and suboptimal results. Deep learning models have, in recent years, been tested as alternative upscaling methods, with promising results (18–20).

This study aimed to investigate the diagnostic accuracy of upscaledMRSI, both compared to and combined with [18F]-FACBC PET, for classifying glioma versus non-tumor tissue and to determine *IDH1* status in patients with gliomas, using image-guided biopsies as reference. The image quality of the low-spatial resolution MRSI data was increased using a deep learning-based model trained to upscale lower-resolution metabolite maps.

## Materials and methods

### Study participants

Twenty-three adult patients undergoing primary resection for diffuse glioma (except one of 23 patient diagnosed with WHO grade 1 ganglioglioma) underwent [18F]-FACBC PET/MRI, including MRSI. At the time of enrollment and imaging, there was no preselection and no prior determination of which patients would undergo image-guided biopsy at locations suitable for spatially matched imaging analysis. Among these, only 13 patients (predominance of high-grade gliomas) ultimately underwent image-guided biopsies with sufficient spatial correspondence and tissue quality to permit reliable correlation with imaging metrics, resulting in the final analytic cohort. Preoperative PET images were fused with FLAIR and ce-T1 MRI using PMOD and imported into the navigation systems (Brainlab or SonoWand). During surgery, intraoperative ultrasound was used to correct for brain shift. Biopsy coordinates were recorded in the ultrasound image space and transformed to the MRI and PET image spaces using the navigation system’s fusion matrices. These coordinates were subsequently mapped to MRSI space via registration to anatomical MRI for metabolite extraction (6). The biopsies were taken from both PET-hotspot and PET-negative areas, when possible (6). All biopsies were sampled from areas with increased FLAIR signal (6).

Three patients were excluded due to loss of image-localization data (n =1) or an uncertain diagnosis based on biopsy (n =2), leaving 10 patients (3 females, aged 24-80) with a total of 32 image-guided biopsies for inclusion in the current study (**Table 1**).

**Table 1:** Summary of patient information.

Patient ID	*IDH1*-mutation status	WHO grade	Glioma subtype	Number of Biopsies	Covered by DL-MRSI	Covered by NN-MRSI	Normal non-tumor
1	*IDH1*-wildtype	4	Glioblastoma	4	4	4	0
2	*IDH1*-wildtype	4	Glioblastoma	4	4	3	1
3	*IDH1*-wildtype	4	Glioblastoma	4	3	2	0
4	*IDH1*-mutated	3	Astrocytoma	4	3	2	0
5	*IDH1*-wildtype	4	Glioblastoma	2	1	1	0
6	*IDH1*-wildtype	4	Glioblastoma	4	4	4	1
7	*IDH1*-wildtype	4	Glioblastoma	1	1	1	0
8	*IDH1*-wildtype	1	Ganglioglioma	4	3	3	4
9	*IDH1*-mutated	3	Astrocytoma	4	4	3	0
10	*IDH1*-mutated	2	Astrocytoma	1	1	1	0

MRSI-DL, upscaling metabolite maps by our deep learning method; MRSI-NN, upscaling by the nearest-neighbor approach.

All participants gave written informed consent. This study was performed in accordance with the Declaration of Helsinki and approved by the Regional Ethics Committee (REK South East Norway, reference number: 2018/2243).

### Biopsy analysis

Twenty-six of the 32 biopsies were classified as glioma tissue (predominance of high-grade gliomas), while six were classified as non-tumor tissue. Patient 8’s tumor (CNS WHO grade 1 ganglioglioma) is not a diffuse glioma under the 2021 WHO classification. Non-tumor tissue (non-neoplastic, FLAIR-abnormal tissue) was defined as normal tissue and/or mild inflammatory changes. The classification was performed by a neuropathologist and was based on immunohistochemical analysis (n =11), DNA methylation analysis (n =10), or both (n =9). The biopsies were also classified as *IDH1*-wildtype (n =8) or *IDH1*-mutated (n =24) as defined by presence/absence of the IDH1 R132H hotspot mutation, and which assay(s), immunohistochemistry, targeted sequencing, and/or DNA methylation profiling, were used to establish it for each biopsy. A more detailed description is provided by Vindstad et al. (6).

### PET/MRI acquisition and reconstruction

Anatomical MRI, proton 2-dimensional MRSI, and [18F]-FACBC PET were acquired using a hybrid PET/MRI system (Siemens Biograph mMR, software version Syngo MR VE11P, Erlangen, Germany). The patients received an injection of [18F]-FACBC (3 ± 0.2 MBq/kg) on the scanner examination table at the onset of the PET/MRI acquisition, and list-mode PET was acquired 0 to 45 min after injection.

Standard MRI sequences were obtained, including pre- and post-contrast-enhanced (ce) 3-dimensional (3D) T1 magnetization prepared for rapid gradient echo imaging (MPRAGE) with TR/TE/TI = 2000/2.43/1100 ms, flip angle of 8°, field of view = 256 × 256 mm^2^, matrix = 256 × 256, slice thickness of 1 mm, nominal voxel size of 1 mm^3^, number of averages of 1, and acquisition time of 4:37 min; and 3D FLAIR 3D FLARE was acquired with TR/TE/TI = 5000/386/1800 ms, flip angle of 120°, FOV of 256×256 mm (2), slice thickness of 1 mm, nominal voxel size of 1 mm^3^, number of average of 1, and total acquisition time of 6:50 min.

MRSI data parameters included a 2-dimensional Point Resolved Spectroscopy (PRESS) sequence, TR/TE = 1700/30 ms, flip angle of 90˚, three averages, matrix size of 16×16, interpolated to 32×32, vector size of 1024, a spectral bandwidth of 1200 Hz, a slice thickness of 16 mm, corresponding to a 6.875×6.875×16 mm^3^ nominal resolution, vendor-provided water suppression CHESS with default values, and a total scan time of 6.8 minutes.

The last 15 minutes of the PET/MRI acquisition were used to reconstruct static PET images. Image reconstruction was performed with an iterative method (3D OSEM algorithm, 3 iterations, 21 subsets, 344×344 matrix, 4 mm Gaussian filter, voxel size: 2.1×2.1×2.0 mm^3^) incorporating point spread function, decay-, scatter-, and attenuation-correction (AC). AC was based on the UTE sequence and the deep learning-based DeepUTE method developed by Ladefoged et al. (21, 22).

### Image and spectra analysis

Metabolite maps were generated by analyzing the MR spectrum from each voxel. The MR spectra from brain voxels were processed with phase and frequency corrections before being analyzed using LCModel Version 6.3 (23) over a range of 0.2 to 4.2 ppm, using a basis-set for the PRESS sequence and an echo time of 30 ms. This basis set encompassed a wide range of metabolites, including aspartate, Cr, gamma-aminobutyric acid, glutamate, glutamine, glutathione, glycine, glycerophosphocholine (GPC), glycerophosphoethanolamine, Myo-inositol, lactate, NAA, glutamate, phosphocholine (PCh), phosphocreatine (PCr), phosphorylethanolamine, scyllo-inositol, serine, glucose, and taurine. Macromolecules and lipids were modeled using LCModel’s default macromolecule basis. For the results, specific metabolite groups were reported as combined measures: tCho as the sum of GPC and PCh, and total creatine (tCr) as the sum of Cr and PCr. To ensure the reliability of the spectral data and metabolite quantification, quality thresholds were applied during LCModel fitting. These criteria included a linewidth of less than 0.15 ppm and a Cramer-Rao Lower Bound (CRLB) below 25% (both linewidth and CRLB were reported by LCModel).

The MRSI-derived metabolite maps were upscaled from 32×32 (in-plane 6.875×6.875 mm^2^) to 128×128 (1.25×1.25 mm^2^), using nearest-neighbor and a trained deep learning model as described previously (24). Custom MATLAB scripts (ver. R2025a, MathWorks, Natick, MA, USA) were used for the upscaling. The deep learning model is trained to better recover anatomical details during the upscaling compared to the alternative methods, facilitating more accurate localization and quantification of metabolic changes, as described in more detail in the **Supplementary Material**. Metabolite ratios, including tCho/NAA and tCho/tCr, were generated and converted into NifTi format. These maps were overlaid on FLAIR and ce-T1 MRI to extract the corresponding voxel values of tCho/NAA and tCho/tCr for each biopsy in FreeView, a viewing platform from FreeSurfer (25). For each biopsy location/voxel, tCho/NAA and tCho/tCr were classified as MRSI positive if the metabolite ratio exceeded the threshold value of tCho/NAA ≥2.37 and tCho/tCr ≥1.21, respectively, based on ROC analysis, with these limits being relatively close to those reported previously indicating an abnormally increased level of choline-containing metabolites (15, 26). Metabolite values were extracted directly from the 1.25×1.25 mm^2^ DL-upscaled grid at the biopsy positions. The MRSI was acquired in a single 2-dimensional slice, covering only parts of the tumor. The number of image-localized biopsies covered by the MRSI slice using the different upscaling methods was therefore evaluated.

Quantitative image analysis in PMOD (version 4.304, PMOD Technologies LLC, Zürich, Switzerland) involved co-registering ce-T1 MRI and PET to the FLAIR images for each individual. [18F]-FACBC uptake was assessed using a spherical 2 mm radius volume of interest (VOI) at the biopsy sites to calculate the mean standardized uptake value (SUV) within the VOI. Tumor-to-background ratios (TBRs) were calculated as the mean SUV at the biopsy site divided by background uptake defined in the contralateral side of the brain (27). The contralateral side of the brain was defined above the ventricles, consisting of six consecutive, crescent-shaped regions of interest, forming a VOI (6). PET volumes were defined using a TBR threshold value of 2.0 (6). PET uptake outside the brain was manually removed. Each biopsy was classified as PET-positive if the biopsy was included in the PET volume (6).

#### Deep learning-based upscaling metabolite maps

The deep learning–based upscaling method used in this study has been previously published by our group (24) and is only summarized here briefly. In short, the model takes low-resolution metabolite maps (32×32), derived from selected tumor-containing T1-weighted and FLAIR slices, and generates corresponding upscaled metabolite maps (128×128). The network is a convolutional architecture incorporating stacked High-Frequency Attention Blocks (HFABs) with skip connections to enhance fine spatial details, particularly at tissue boundaries. A gated feed-forward network is used to improve feature refinement and stability. The model was trained on synthetic metabolite maps paired with FLAIR images and optimized to super-resolve key tumor-related metabolites and ratios (e.g., tCho, tCr, tNAA (NAA+NAAG), tCho/NAA, tCho/tCr). Full architectural and experimental details are provided elsewhere (24).

### Statistical analysis

Statistical analysis was performed in Python using custom scripts. Fisher’s exact test was used for categorical variables (e.g., biopsy covered/not covered by MRSI) to evaluate statistical significance. Receiver operating characteristic (ROC) curve analysis assessed the diagnostic accuracy of tCho/NAA, tCho/tCr, TBR, SUV, and combinations of the modalities for classifying glioma vs. non-tumor tissue and *IDH1*-mutation status. AUC discrimination was classified as follows: <0.5 (none), 0.5–0.7 (poor), 0.7–0.8 (acceptable), 0.8–0.9 (excellent), and ≥0.9 (outstanding) (28). Optimal thresholds were defined as the ROC curve point closest to the top-left corner. The DeLong test was used to compare the ROC curves and evaluate statistical differences in their performance as an exploratory approach. Because the DeLong test for correlated ROC curves requires that all curves being compared derive from the same set of observations, all DeLong comparisons involving MRSI-derived parameters (tCho/NAA, tCho/tCr) were restricted to the subset of biopsies with mutual PET and MRSI coverage (e.g., n=29 for glioma vs. non-tumor classification); SUV and TBR values entering these specific comparisons were therefore recomputed on this same n=29 subsample, distinct from the full n=32 cohort used for PET-only analyses. The classification performance of the PET parameters SUV and TBR, and MRSI-derived tCho/NAA and tCho/tCr was investigated as stand-alone parameters, as well as for each combination; e.g., tCho/NAA + SUV, tCho/tCr + TBR. To evaluate the combined classification performance of the combined variables, we fitted a logistic regression model using both variables as predictors. The model estimated predicted probabilities for the outcome, which were then used in an ROC analysis. This approach enabled the assessment of the combined discriminative power of the two variables. We compared the AUC of this combined model to those from the ROC analyses of the individual variables to evaluate improvement in classification performance. When using predicted probabilities from regression in ROC analysis, the thresholds are typically between 0 and 1. These represent decision cutoffs on the probability scale for classifying an observation as positive or negative.

We also investigated how accurately these stand-alone and combined parameters classify glioma from non-tumor tissue by calculating their confusion matrices, using the optimal thresholds derived from the ROC analysis.

## Results

### Biopsy coverage

While the DL-based maps still covered a smaller part of the tumor and thus fewer biopsies than PET (12.5% of total biopsies are not covered by DL-based maps, *p* = 0.06), they significantly improved coverage compared to the nearest-neighbor method (25%, *p* = 0.005) (**Figure 1**). Also, **Figure 1** shows MRSI data in one hemisphere data, as 2D MRSI does not cover the entire brain. Even though the original MRSI acquisition covered a 220 × 220 mm^2^ FOV, the reconstructed higher-resolution maps are restricted to a smaller effective region (128 × 128 matrix at 1.25 mm resolution; ~160 × 160 mm^2^), corresponding to the shimmed spectroscopy volume. Signal outside this region was suppressed by outer volume suppression and therefore does not contain usable MRS data. More representative axial views illustrating biopsy positions in relation to multimodal PET-MRI modalities are given in **Figure S1**.

**Figure 1:**
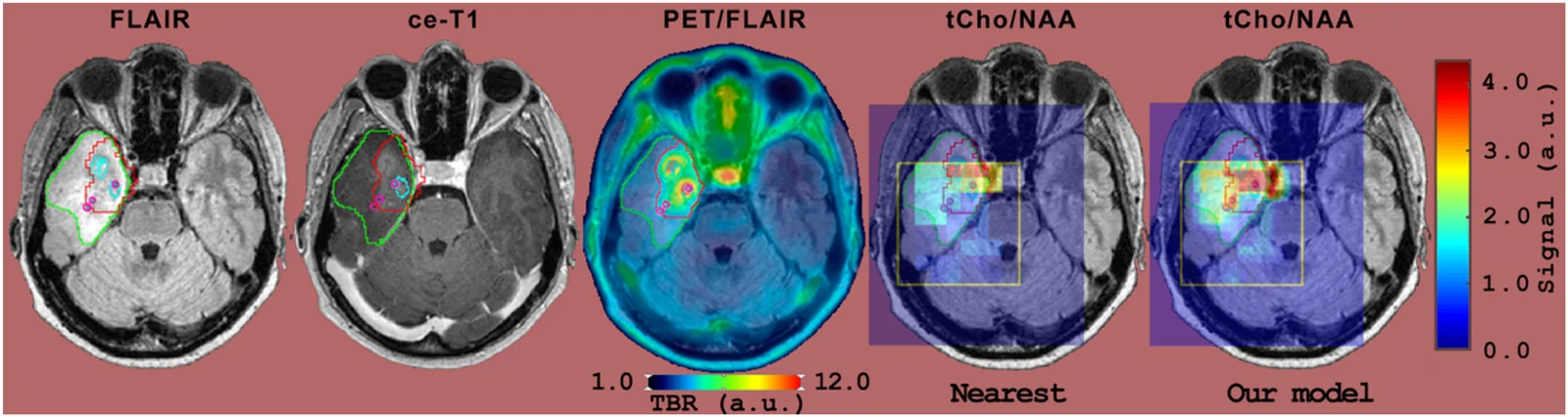
An example axial view from left to right: fluid-attenuated inversion recovery (FLAIR), contrast-enhanced T1-weighted (ce-T1), combined FLAIR/PET, and upscaled total-Choline/N-acetyl aspartate (tCho/NAA) maps using nearest-neighbor interpolation and the DL-model for a patient with glioma. Colored contours indicate tumor area/volume determined by FLAIR (green), ce-T1 (cyan), TBR ≥2.0 (red, PET image), and tCho/NAA ≥2.37 (red). Blue circles indicate biopsy sites classified as high-grade glioma. Notably, two biopsies lacked contrast enhancement but exhibited [18F]-FACBC uptake ≥2.0. One biopsy is outside the margin determined by the tCho/NAA map upscaled using the nearest-neighbor method. In contrast, all biopsies (magenta circles, rightmost panel) are included within the tumor area/volume determined using DL model upscaling. Nevertheless, the MRSI acquisition slab did not cover one biopsy for this patient (Patient 3, **Table 1**). Metabolite maps were acquired within the yellow box, and the blue regions were suppressed by outer-volume suppression. Additional voxels without displayed data were excluded based on spectral quality criteria.

### Classification of glioma vs non-tumor tissue

In the ROC analysis, the DL-based MRS showed higher apparent diagnostic accuracy than nearest-neighbor interpolation, with AUCs of 0.87 (95% CI: 0.66–1.00) and 0.81 (95% CI: 0.54–1.00) versus 0.55 (95% CI: 0.26–0.82) and 0.62 (95% CI: 0.36–0.87) for tCho/NAA and tCho/tCr, respectively (*p* = 0.016 and *p* = 0.033, given the small, within-patient-clustered sample and uncorrected multiple comparisons, these p-values should be interpreted with caution and the apparent difference regarded as exploratory rather than confirmatory). Further results are therefore presented only for the DL-based MRSI maps.The results from the ROC analysis of glioma/non-tumor classification of the biopsies are presented in **Figure 2**. The performance of the tCho/NAA and tCho/tCr ratios is shown in **Figure 2**, demonstrating their distinct diagnostic accuracy. However, tCho/tCr exhibited the highest sensitivity (0.92). SUV had acceptable performance (AUC = 0.71, 95% CI: 0.49-0.90), and TBR had poor performance (AUC = 0.68, 95% CI: 0.46-0.86). All values are listed in **Supplementary Table S1**.

**Figure 2:**
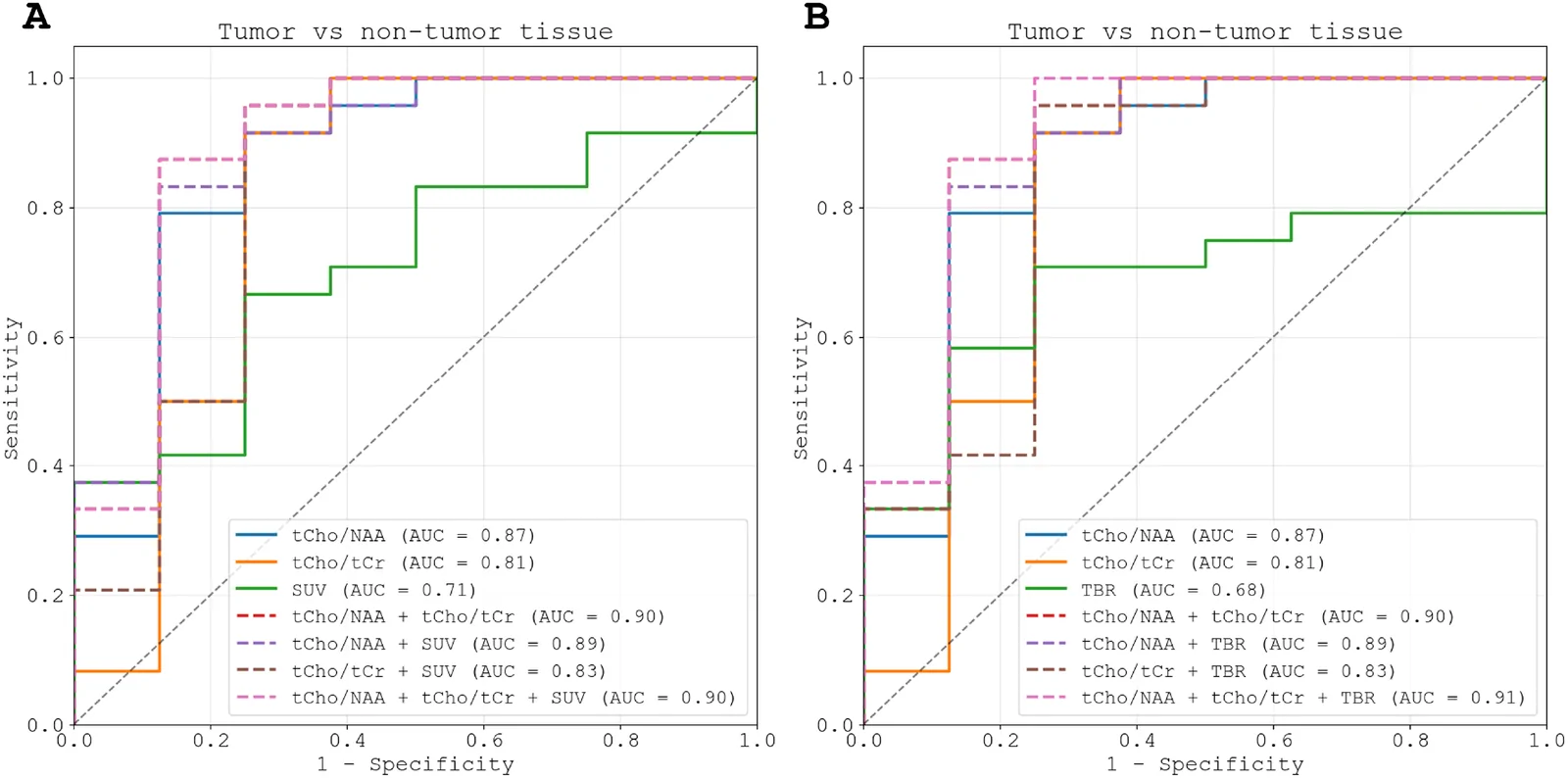
Receiver operating characteristic (ROC) curve analysis was performed as a measure of the diagnostic accuracy of glioma (n =23 biopsies) vs. non-tumor (n =6 biopsies) tissue classification for tCho/NAA (n =29), tCho/tCr (n =29), SUV (n =32) **(A)**, tumor-to-background ratio (TBR, n=32) **(B)**, and a combination of all covariates in the discrimination of glioma from non-tumor tissues (n =29). The MRSI-derived metabolite ratios are from the deep-learning upscaled MRSI maps. Classification performance was quantified using area under the ROC curve (AUC), and statistical differences between ROC curves were assessed using the DeLong test for correlated ROC curves (*p* < 0.05).

The combination of TBR or SUV with tCho/NAA or tCho/tCr improved the glioma/non-tumor classification compared to either modality alone, as demonstrated with the ROC curves in **Figure 2** (**Supplementary Table S1**) and the confusion matrix in **Figure 3**. ROC analysis identified an optimal threshold of tCho/NAA ≥2.37 and tCho/tCr ≥1.21 for distinguishing between groups. The optimal combination, tCho/NAA + tCho/tCr + TBR, achieved an AUC of 0.91 (95% CI: 0.71-1.00). However, the heatmap of the DeLong analysis in **Figure 4** shows that only the combinations including TBR, tCho/NAA, and/or tCho/tCr provide significantly higher diagnostic accuracy than the individual modalities (*p* < 0.05, log10(*p*) >1.3) (the *p*-values from the DeLong test were not corrected).

**Figure 3:**
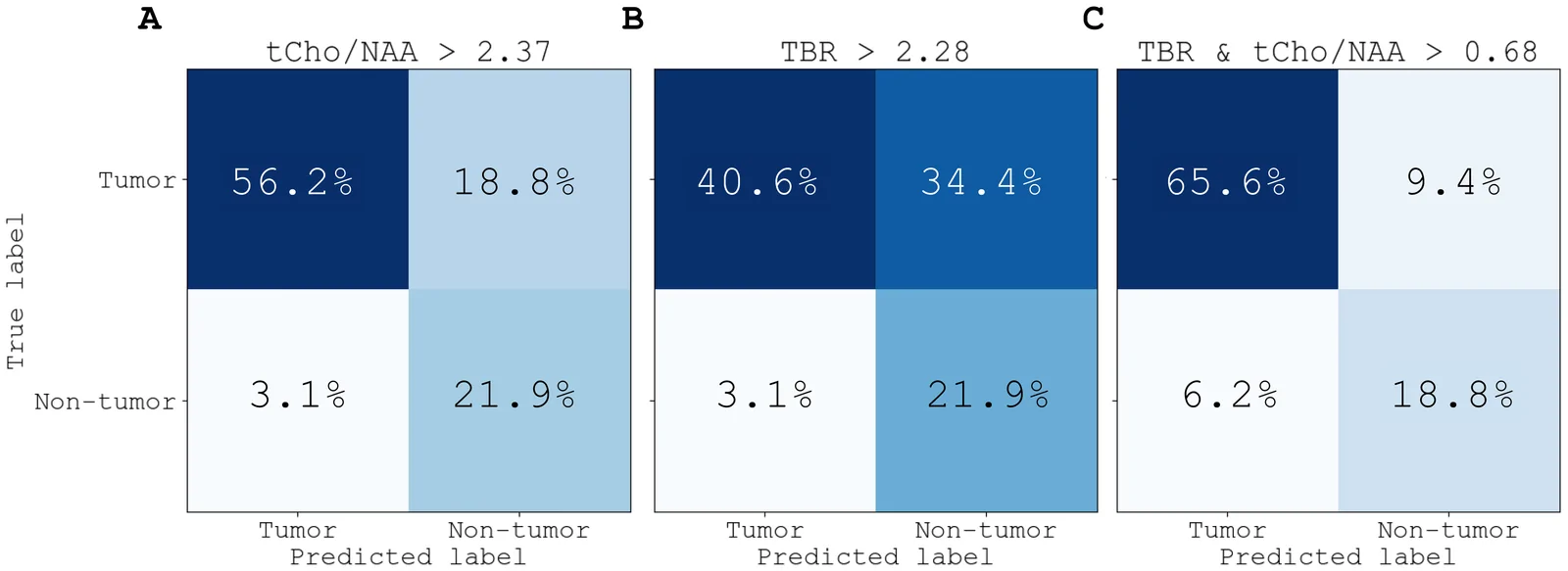
Confusion matrix derived using the optimal classification threshold determined from ROC-analysis for **(A)** tCho/NAA (n =29), **(B)** tumor-to-background ratio (TBR, n=32), and **(C)** the combined modality of TBR and tCho/NAA (n =29) for discrimination of glioma vs. non-tumor tissue (As PET-MRSI mutual coverage, n =29 biopsies). ROC analysis was used to determine optimal thresholds (tCho/NAA ≥2.37; tCho/tCr ≥1.21). The combined model (tCho/NAA + tCho/tCr + TBR) achieved an AUC of 0.91 (95% CI: 0.71–1.00, *p* = 0.0003). MRSI-derived metabolic ratios were calculated from deep learning-upscaled MRSI maps. The experimental unit for all analyses was the individual biopsy, with one classification outcome per subject.

**Figure 4:**
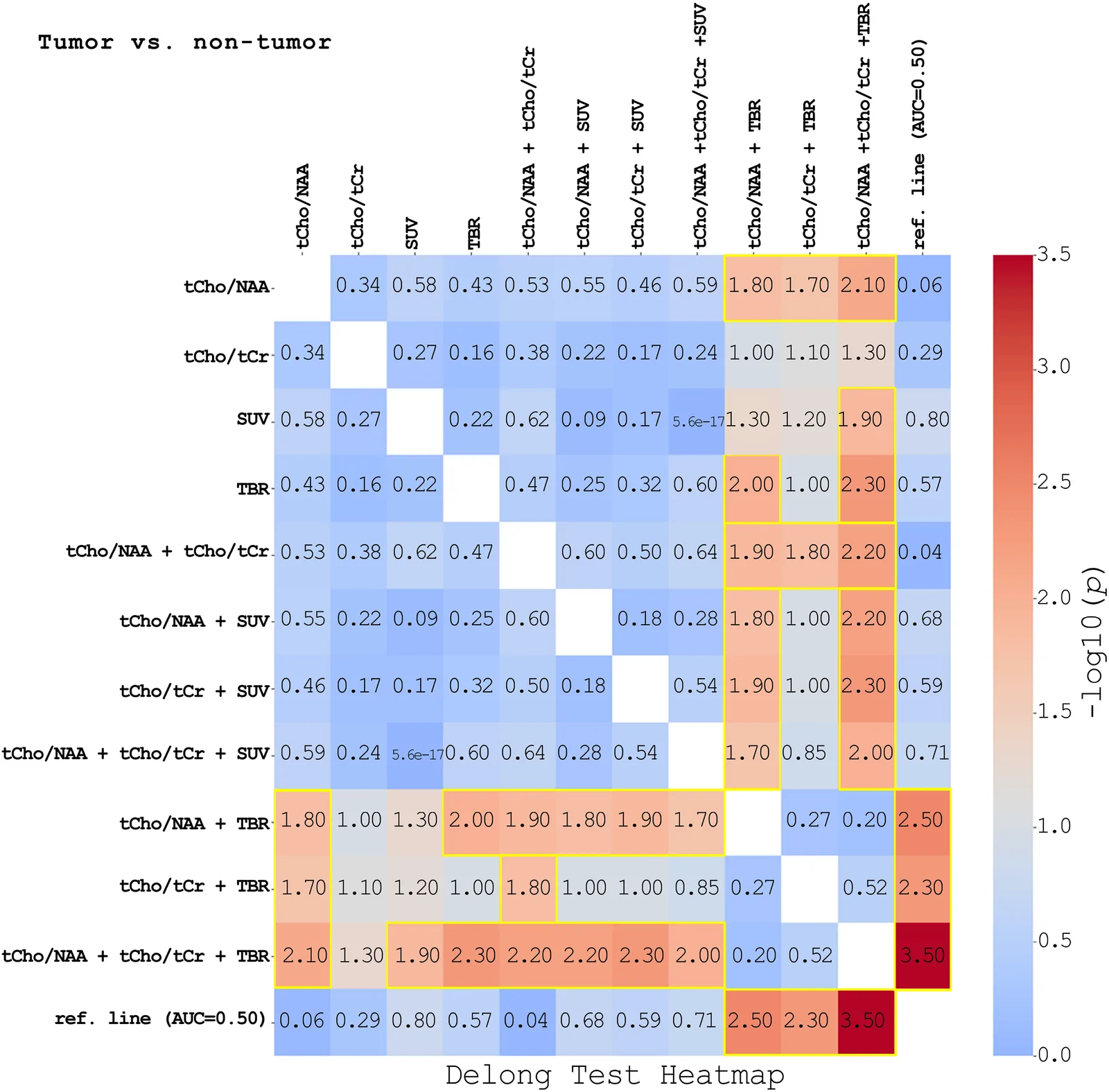
Heatmap showing the result of the DeLong test to evaluate statistical significance among ROC curves assessing the diagnostic accuracy of glioma vs. non-tumor tissue classification [curves for tCho/NAA (n =29), tCho/tCr (n =29), SUV (n =32), tumor-to-background ratio (TBR, n=32), and the combination of them (n =29)]. Results are listed as -log10 (*p*-values), with values >1.3 (light orange to dark red) indicating statistical significance. MRSI-derived metabolic ratios are calculated using the deep learning-upscaled MRSI maps. *P*-values from the DeLong test were not corrected for multiple comparisons and should therefore be interpreted as exploratory.

### Classification of *IDH1* mutation status

The results from the ROC analysis of *IDH1* mutation status of the biopsies are presented in **Figure 5**. The metabolite ratios tCho/NAA and tCho/tCr show poor performance for classifying *IDH1*-mutation status (AUC = 0.67, 95% CI: 0.44-0.88 and AUC = 0.38, 95% CI: 0.17-0.60). TBR and SUV both demonstrated stronger performance, with SUV being slightly better, with an AUC of 0.83 (95% CI: 0.66-0.95) for *IDH1* status. The combination of SUV, tCho/NAA, and tCho/tCr improved accuracy for classification of *IDH1* mutation status (AUC = 0.96, 95% CI: 0.88-1.00, *p* < 0.05) compared to standalone metabolite ratios (**Figures 5**, **6**; **Supplementary Table S2**).

**Figure 5:**
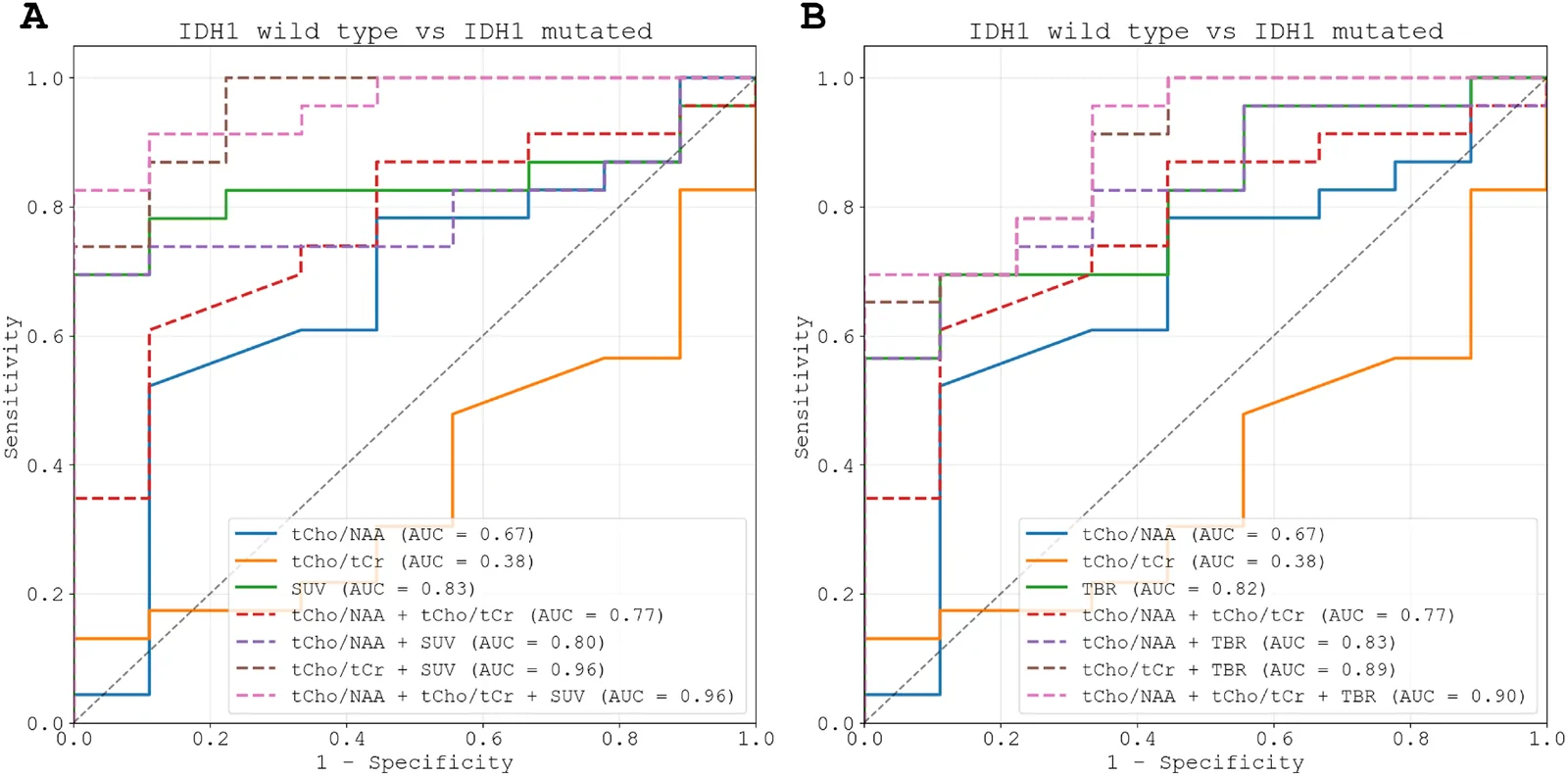
Receiver operating characteristic (ROC) curves for MRSI-derived metabolite ratios (tCho/NAA, tCho/tCr) (n =29) and SUV (n =32) **(A)** or tumor-to-background ratio (TBR, n=32) **(B)**, and a selective combination of covariates when applied to discrimination of IDH1-wildtype from IDH1-mutated tissue. Metabolic ratios were calculated from deep learning-upscaled MRSI maps. The experimental unit for all analyses was the individual biopsy, with one value per metric per subject. Classification performance was quantified using the area under the ROC curve (AUC), and statistical differences between ROC curves were assessed using the DeLong test for correlated ROC curves (*p* < 0.05).

**Figure 6:**
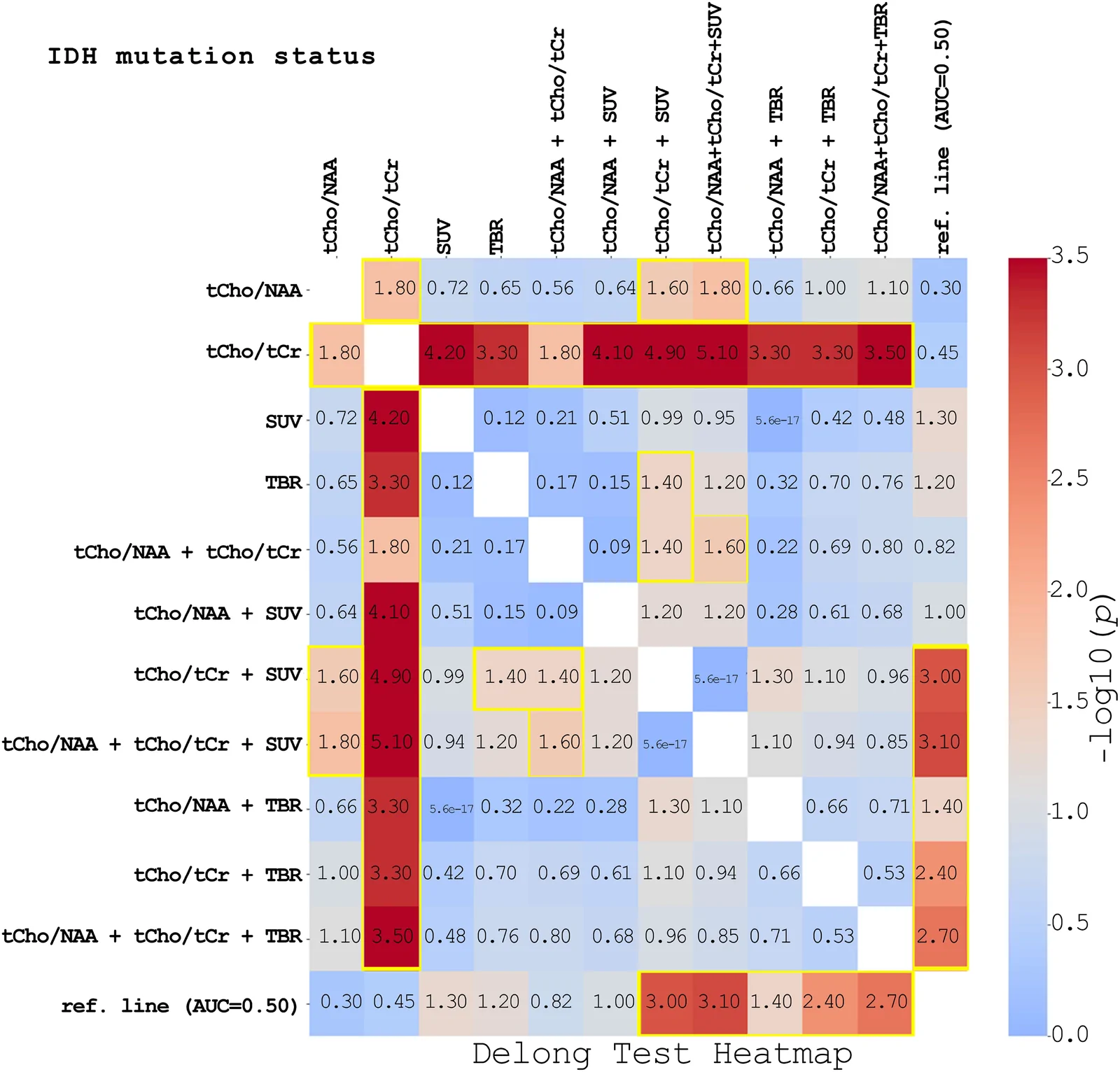
Heatmap summarizing pairwise statistical comparisons between receiver operating characteristic (ROC) curves for the classification of IDH1 mutation status (curves for tCho/NAA (n =29), tCho/tCr (n =29), SUV (n =32), tumor-to-background ratio (TBR, n=32), and the combination of them (n =29)) Statistical significance was assessed using the DeLong test for correlated ROC curves, and results are displayed as -log10 (p-values), with values >1.3 (light orange to dark red) indicating significance (*p* < 0.05). MRSI-derived metabolic ratios were computed from DL-upscaled MRSI maps. The experimental unit for all analyses was the individual biopsy, with one ROC curve generated per imaging metric. *P*-values from the DeLong test were not corrected for multiple comparisons and should therefore be interpreted as exploratory.

## Discussion

The results presented in this exploratory study highlight the diagnostic potential of metabolic ratios derived from MRSI and suggest that tCho/NAA and tCho/tCr can improve diagnostic accuracy beyond [18F]-FACBC PET and MRSI alone. A combination of TBR, tCho/NAA, and tCho/tCr yielded the highest AUC (AUC = 0.91, *p* < 0.05) for classification of glioma tissue versus non-tumor tissue, while combining tCho/NAA, tCho/tCr, and SUV improved the classification of *IDH1*-wildtype and *IDH1*-mutated tissues (AUC = 0.96, *p* < 0.05) compared to the standalone metabolite ratios. However, since these findings are based on biopsies rather than a patient-level analysis, the results should be interpreted cautiously.

MRSI did not cover the entire tumor and was not applicable for calculation in all biopsies. In this study, two-dimensional MRSI slices were selectively placed in a region covering a larger area of the lesion, successfully identifying parts of the tumor in most subjects. The time window for MRSI examination is extremely limited when acquired after the other MR sequences, such as fluid-attenuated inversion recovery (FLAIR), T1W (with/without contrast), T2W, DWI, perfusion, and potentially functional MRI. Thus, the choice of slab MRSI acquisition over whole-brain MRSI was due to the longer scan time of the vendor-provided 3D PRESS sequence, which is approximately 20 minutes (29). The use of recently developed faster 3D MRSI techniques, with scan times under seven minutes, enables whole-brain metabolite imaging, allowing comprehensive lesion coverage (17, 30, 31).

An optimal outcome of the upscaling method would be an improved effective spatial resolution, enabling more detailed visualization of structures, particularly in areas that are typically difficult to distinguish, such as edges (32–34). The DL method improves the effective spatial resolution markedly by learning to fill in more accurate and detailed information, especially around edges and boundaries (32), which would be missed by traditional methods such as nearest-neighbor and spline interpolation. The improved performance of the DL method may be attributed to the incorporation of prior anatomical knowledge, allowing for more precise metabolic mapping and increased alignment with tumor regions, especially in complex, heterogeneous gliomas, as demonstrated in previous studies (19, 20). Given the limited number of patients in this study, we cannot yet conclude the utility of the DL method in the precise diagnosis of gliomas, but our results suggest its potential.

Compared to normal healthy brain tissue, increased levels of tCho/NAA and tCho/tCr have been previously reported in malignant gliomas (15, 16, 35). In this study, tCho/NAA was the most accurate modality, with an AUC of 0.87 in differentiating glioma from non-tumor tissue. Despite this, tCho/tCr had the highest sensitivity (0.92), suggesting that this parameter might be a valuable diagnostic tool for detecting subtle metabolic changes, though with a lower specificity than tCho/NAA. TBR showed poor and SUV acceptable performance according to AUC measures, which may indicate limited diagnostic utility as standalone measures, at least in our cohort. However, it is important to acknowledge that comparisons between PET and MRSI are influenced by the imaging method used for biopsy selection, since PET and MRSI show different metabolic changes. Compared to our results, a previous study reported an opposite trend, where amino acid PET using [18F]-FET yielded higher AUC values than MRSI (36). In their study, due to the lack of MRSI data at the time of biopsy, trajectories were planned based on PET, targeting the most metabolically active tumor regions. This study design, therefore, inherently favored [18F]-FET PET over MRSI. However, previous studies (26, 27) have demonstrated the potential utility of MRSI in biopsy planning, suggesting that a prospective study incorporating both modalities could provide a more balanced and comprehensive comparison. Another major contributing factor for discrepancies in reported diagnostic performance is threshold determination for SUV, which varies across studies and is influenced by factors such as the amino PET tracer used, PET scanner differences, reconstruction variations, and patient selection criteria. As previous research has shown, these parameters can cause slight shifts in threshold values, highlighting the need for larger, biopsy-controlled studies to establish and validate optimal cutoff values.

Gliomas are heterogeneous, with regions of varying activity and molecular properties, complicating diagnosis and the identification of aggressive tumor areas (3). Compared to PET/CT, MRSI combined with amino acid PET has been shown to enhance diagnostic accuracy for patients with gliomas by improving tumor detection, characterizing heterogeneity, and assessing treatment responses more effectively (37–41). Furthermore, a prospective study of 50 glioma patients by Floeth et al. (42) demonstrated that integrating [18F]-FET-PET and MRS significantly improved diagnostic accuracy, though we note that the study’s substantially larger, prospective design differs from the present pilot cohort, and similar validation will be needed here before comparable claims can be made. When both modalities were positive, histological confirmation of tumors reached 97%, whereas negative findings consistently indicated nonneoplastic disease. These results underscore the added value of PET and MRSI in cases where MRI alone is inconclusive (43). The increased diagnostic accuracy could be caused by the fact that the two methods can provide complementary information, as PET primarily reflects specific metabolic pathways, while MRSI provides biochemical concentrations within the tumor (43–45).

As amino acid PET imaging becomes more widely available, it is increasingly recognized as a reliable marker of glioma infiltration and IDH status. [18F]-FET PET has shown promise in distinguishing *IDH1*-wildtype from *IDH1*-mutated gliomas by leveraging metabolic differences, aiding diagnosis and treatment planning (46–50). Studies using TBR demonstrated their effectiveness in differentiating molecular subtypes, aligning with our findings and highlighting the value of amino acid PET-derived parameters like SUVs and TBRs for noninvasive IDH status characterization. However, none of these studies incorporated MRSI data, limiting direct comparison with our results on combined PET/MRSI modalities for identifying *IDH1*-mutated gliomas. Recent studies have demonstrated the utility of [18F]-FET PET (51) and emerging PET tracers (52, 53) targeting *IDH* mutations, suggesting a potential paradigm shift in the diagnosis of gliomas and treatment planning.

One limitation of the study is the relatively small sample size. Thus, the reported AUCs are apparent (resubstitution) estimates from a small and unbalanced sample; no internal or external validation was feasible given the cohort size; and the values should be interpreted as exploratory/hypothesis-generating rather than validated diagnostic performance, pending replication in a larger, prospectively designed cohort with better-balanced non-tumor sampling. Most of the tumors are of a higher grade, and the results of this study might not be transferable to low-grade samples and less common glioma types. Another limitation is the stratification task, which was limited to tumor and non-tumor tissue, and necrotic tissue was included within the tumor class due to the small sample size. Some variation in our results may arise from differences in spatial resolution, inherent biological differences between [18F]-FACBC uptake and MRS metabolites, and the small sample size with unbalanced classes. Also, the nominal resolutions of MRSI (10×10×10 mm^3^, upscaled to 1.25×1.25×10 mm^3^ using DL upscaling) and [18F]-FACBC PET (reconstructed to 2.1×2.1×2.0 mm^3^ with PSF modeling and smoothing) differ substantially, resulting in distinct spatial averaging and potential artifacts. Biopsies were small (~1 mm cores) and co-registered to ce-T1 and FLAIR modalities, but partial volume effects and resolution mismatches may contribute to variability in measured signals. PET uptake was quantified using a 2-mm-radius VOI to account for biopsy uncertainty and PET resolution. MRSI values were extracted at corresponding locations, reflecting the native MRSI voxel size. Due to fundamentally different spatial resolutions, identical VOI sizes were not applied across modalities. Thus, a limitation of this study is the use of modality-specific spatial averaging strategies for PET and MRSI. While PET values were extracted using a 2-mm-radius VOI to account for spatial uncertainty and resolution, MRSI-derived tCho/NAA values inherently reflect averaging at the voxel level. These differences may affect the direct comparability of quantitative measures between modalities. These factors should be considered when interpreting the comparative accuracy of MRSI and PET and represent inherent limitations of multimodal analysis in this study. In addition, DL-based upscaling was not applied to PET data, as PET already benefits from established reconstruction methods, and the DL model used here was specifically trained on MRSI-derived metabolite maps combined with anatomical MR images; extending this approach to PET would require a dedicated PET-specific model and represents an avenue for future work. Acquiring simultaneous PET and MRSI remains challenging and limited the cohort size; nevertheless, PET demonstrated high sensitivity, and MRSI showed robust performance for the major metabolites analyzed, with low variability and statistically significant results. The predominance of high-grade gliomas and unbalanced class sizes limits generalizability, and results should therefore be interpreted within this clinical context.

The DL-based upscaling model (24) employed in this study was trained on the BraTS2020 dataset, in which tumor masks were derived from conventional MRI modalities rather than spectroscopic data. As metabolite signal distributions only partially overlap with MRI-defined tumor boundaries, this domain mismatch may limit the model’s upscaling performance for real clinical MRSI data, and the observed improvement over nearest-neighbor interpolation should be interpreted cautiously. Additionally, synthetic lesions were assigned uniform metabolite concentrations within each tissue compartment, whereas real gliomas exhibit substantial intra-tumoral heterogeneity reflecting necrosis, hypoxia, and variable proliferative activity. Also, aggregate evaluation metrics reported in (24) were computed over full volumes, where normal brain tissue constitutes most voxels. As upscaling in normal tissue is comparatively straightforward, these metrics may overestimate model performance in tumor regions. Future validation should report tumor-mask-specific metrics to more meaningfully reflect clinical utility. Future work should prioritize retraining and validating the model using larger cohorts of real clinical MRSI data to establish its generalizability across glioma grades and acquisition protocols.

Gliomas are heterogeneous tumors; however, in this study, PET and MRSI outcomes were analyzed based strictly on biopsy locations, extracting metabolite ratios and PET-derived parameters only for these regions. There is also a lack of biopsies from normal tissue to be used as “control”, as biopsies were only targeting the tumors according to increased FLAIR signal. This might affect the calculation of specificity, due to an imbalance in the dataset. Another notable effect is that contrast enhancement may influence MRSI signals, for example, by altering relaxation times or local magnetic susceptibility (54). Furthermore, our deep learning model, which was used to upscale the MRSI data, was trained on synthetic datasets. This could limit its generalizability, as it does not account for several challenging factors, such as eddy current artifacts, field inhomogeneity, variable acquisitions, and MR system setups. Additionally, the inherent black-box nature of deep learning models raises concerns regarding ethical issues and risk management due to the lack of interpretability and, by extension, the trustworthiness of their outputs (55, 56).

Future works may focus on addressing the study’s limitations, such as the small sample size, by expanding the cohort to include low-grade gliomas. Additionally, training deep learning models on *in vivo* datasets with varied artifacts and MR setups could enhance model generalizability. Exploring multi-modal imaging and improving the interpretability of AI models will also be important for addressing ethical concerns and improving clinical trust. These efforts could advance AI-powered glioma precision medicine.

## Conclusion

The deep learning-based MRSI model using the tCho/NAA ratio outperformed SUV, TBR, and tCho/tCr in diagnosing glioma versus non-tumor regions, while combining TBR with tCho/NAA and/or tCho/tCr improved tissue classification compared to either modality alone. For distinguishing *IDH1*-mutation status, SUV and TBR outperformed the MRSI-derived parameters, while a combination of tCho/tCr with TBR and SUV slightly improved the classification. Our results may reinforce the notion that PET and MRSI provide complementary insights that can potentially enhance diagnosis. Future studies with larger, biopsy-controlled cohorts and patient-level statistical analysis are needed to refine the integrated PET/MRSI approach, account for within-patient clustering of biopsies, and validate its combined clinical impact. Standardizing acquisition protocols and threshold values across different PET tracers and scanners will also be crucial in improving reproducibility and reliability in glioma diagnostics.

## Data Availability

The original contributions presented in the study are included in the article/**Supplementary Material**. Further inquiries can be directed to the corresponding author.
