# Supplementary material for: A biopsy-validated study of combined DL-upscaled MRSI and (18F)-FACBC PET for presurgical glioma diagnosis
Source: Front Oncol. 2026 Sep 30;16:1968798. doi: 10.3389/fonc.2026.1968798 (PMC13661501; doi:10.3389/fonc.2026.1968798)
Supplement: Supplementary file 1 [file DataSheet1.pdf]

Biopsy-validated study of combined DL-upscaled MRSI and (18F)-FACBC PET for presurgical glioma diagnosis

Bjørkeli EB et al.

Table S1. Results from ROC analysis for tCho/NAA, tCho/tCr, SUV, and TBR for classification of glioma versus non-tumor tissue. Sensitivity, specificity, optimal threshold, and AUC with 95% CI are listed. MRSI-derived metabolic ratios were calculated using the deep learning model.

	Deep Learning model			
	**Sens.**	**Spes.**	**Threshold**	**AUC (95% CI)**
tCho/NAA	0.79	0.88	2.37	0.87 (0.66-1.00)
tCho/tCr	0.92	0.75	1.21	0.81 (0.54-1.00)
SUV	0.67	0.75	0.55	0.71 (0.49-0.90)
TBR	0.58	0.88	2.28	0.68 (0.48-0.86)
tCho/NAA + tCho/tCr	0.88	0.88	0.80	0.90 (0.69-1.00)
tCho/NAA + SUV	0.83	0.88	0.76	0.89 (0.70-1.00)
tCho/NAA + TBR	0.83	0.88	0.76	0.89 (0.70-1.00)
tCho/tCr + SUV	0.95	0.74	0.60	0.83 (0.59-1.00)
tCho/tCr + TBR	0.96	0.75	0.61	0.83 (0.59-1.00)
tCho/NAA + tCho/tCr + SUV	0.88	0.88	0.78	0.90 (0.69-1.00)
tCho/NAA + tCho/tCr + TBR	0.88	0.88	0.77	0.91 (0.71-1.00)

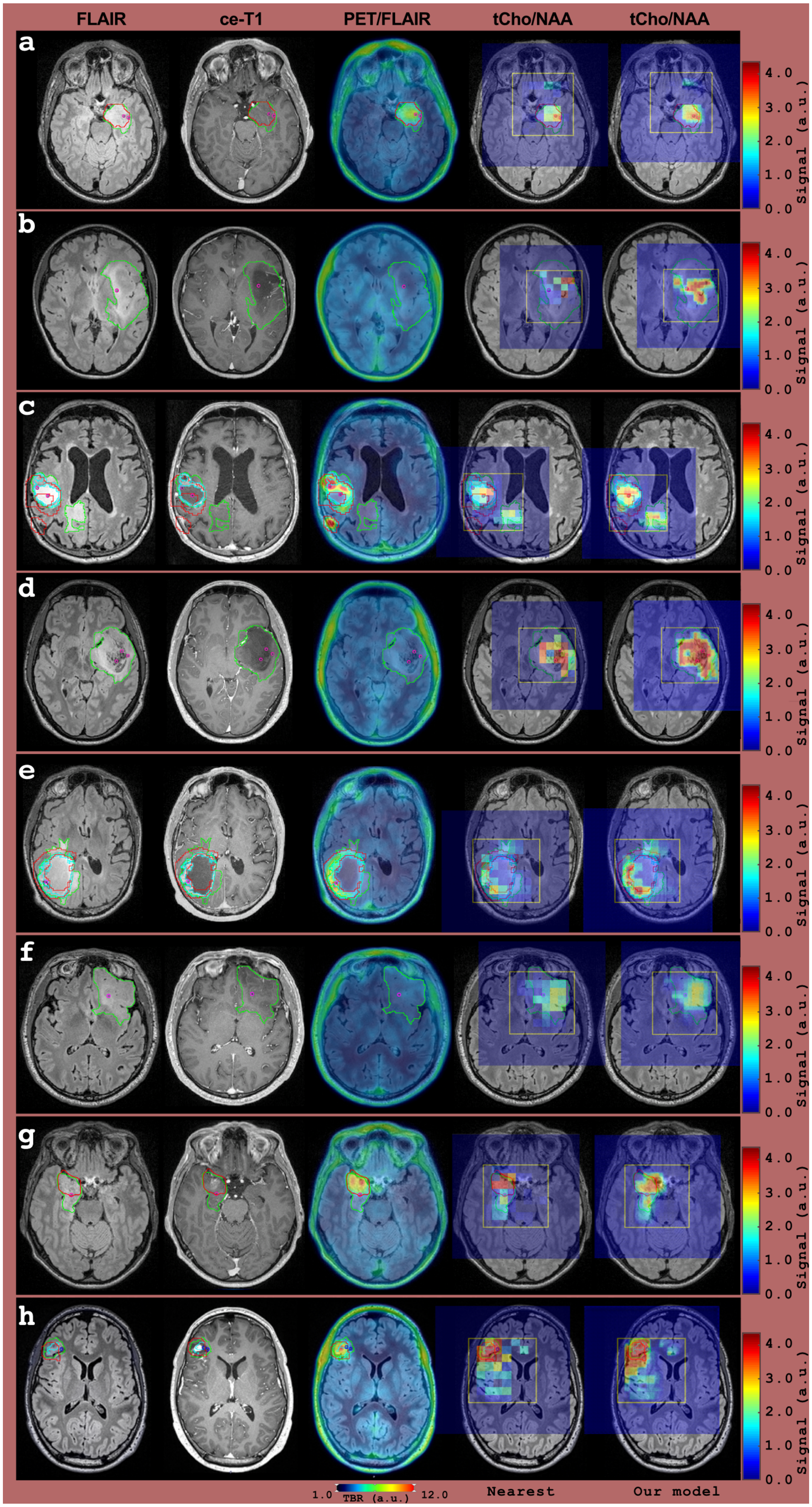

FLAIR
ce-T1
PET/FLAIR
tCho/NAA
tCho/NAA
a
b
c
d
e
f
g
h
4.0
3.0
2.0
1.0
0.0
Signal (a.u.)
1.0
TBR (a.u.)
12.0
Nearest
Our model

Figure S1. Representative axial views illustrating biopsy positions in relation to multimodal PET-MRI modalities across eight patients (five glioblastoma, three lower-grade glioma). Rows (**a–h**) correspond to individual patients. Columns display, from left to right: fluid-attenuated inversion recovery (FLAIR), contrast-enhanced T1-weighted (ce-T1), a combined FLAIR/PET overlay, total-choline-to-N-acetylaspartate ratio (tCho/NAA) maps upscaled using nearest-neighbor interpolation (tCho/NAA—Nearest), and tCho/NAA maps upscaled using our deep learning model. Colored contours delineate tumor extent as defined by FLAIR (green), ce-T1 (cyan), and a tumor-to-background ratio (TBR) ≥ 2.0 on [^{18}F]-FACBC PET (red). Metabolic tumor burden on MRSI maps was defined by a tCho/NAA threshold ≥ 2.37. Magenta circles indicate biopsy sites. Patients 4, 9, and 10 (rows **b**, **d**, and **f**; Table 1) represent lower-grade gliomas that were delineated by FLAIR and ce-T1 but showed absent or limited [^{18}F]-FACBC PET uptake (TBR < 2.0), consistent with the characteristically lower amino acid avidity of lower-grade tumors. The tCho/NAA maps for these cases exhibited mixed signal intensities under both upscaling methods. Regarding spatial concordance between biopsy sites and metabolic tumor extent: a few biopsy sites fall outside the tumor margin as defined by the nearest-neighbor-upscaled tCho/NAA map, whereas most of the biopsy sites fall within the tumor region defined by the DL-upscaled tCho/NAA map (e.g., **b** & **c**). Metabolite maps were acquired within the region indicated by the yellow box. Voxels outside this region, or those failing spectral quality criteria, are not displayed.

Table S2. Results from ROC analysis for tCho/NAA, tCho/tCr, SUV, and TBR for classification of *IDH1*-wildtype from *IDH1*-mutated tissue. Sensitivity, specificity, optimal threshold, and AUC with 95% CI are listed.

	Deep Learning model			
	**Sens.**	**Spes.**	**Threshold**	**AUC (95% CI)**
tCho/NAA	0.52	0.89	2.55	0.67 (0.44-0.88)
tCho/tCr	0.13	1.0	2.79	0.38 (0.17-0.60)
SUV	0.70	1.0	0.73	0.83 (0.66-0.95)
TBR	0.70	0.89	2.06	0.82 (0.65-0.94)
tCho/NAA + tCho/tCr	0.61	0.89	0.74	0.77 (0.58-0.92)
tCho/NAA + SUV	0.70	1.00	0.68	0.80 (0.62-0.93)
tCho/NAA + TBR	0.70	0.89	0.69	0.83 (0.67-0.96)
tCho/tCr + SUV	1.00	0.78	0.56	0.95 (0.87-1.00)
tCho/tCr + TBR	0.65	1.00	0.82	0.89 (0.76-0.99)
tCho/NAA + tCho/tCr + SUV	1.00	0.83	0.70	0.96 (0.88-1.00)
tCho/NAA + tCho/tCr + TBR	0.70	1.00	0.77	0.90 (0.78-1.00)
